# The role of nuclear cardiac imaging in redefining neurogenic stunned myocardium in subarachnoid hemorrhage: a deeper look into the heart

**DOI:** 10.1186/s13054-014-0490-4

**Published:** 2014-08-27

**Authors:** John Papanikolaou, Demosthenes Makris, Epaminondas Zakynthinos

**Affiliations:** Department of Critical Care, School of Medicine, University of Thessaly, University Hospital of Larissa, Biopolis, Larissa, Thessaly 41110 Greece

## Abstract

Subarachnoid hemorrhage may be complicated by neurogenic stunned myocardium, a catecholamine-induced transient cardiomyopathy that displays a wide clinical spectrum of cardiac abnormalities, including electrocardiographic changes, arrhythmias, myocardial necrosis, and left ventricular systolic and diastolic dysfunction. However, less is known about the cardiac metabolic consequences of acute subarachnoid hemorrhage. Prunet and coworkers’ recent study provides scintigraphic evidence suggesting that glucose metabolism and sympathetic cardiac innervation are severely and globally depressed during the acute phase of the disease. Metabolic and innervation abnormalities are largely overlapped and are probably not causally related to myocardial ischemia, suggesting that impaired glucose metabolism is probably neurogenic in nature. The scintigraphic defects seem to reverse slowly, within months of the onset of cerebral bleeding. Interestingly, scintigraphic evidence of metabolic myocardial alterations may exist even in the absence of clinical features of cardiac disease, possibly representing a subclinical type of neurogenic stunned myocardium.

Subarachnoid hemorrhage (SAH) may cause transient cardiac dysfunction, possibly attributed to massive release of catecholamines. The influence of catecholamine oversecretion on cardiac metabolism has been extensively studied in Takotsubo cardiomyopathy (TTC), which is also considered catecholamine induced in nature. However, data regarding acute myocardial metabolic alterations in SAH are sparse. The recent article by Prunet and colleagues in *Critical Care* deals with this poorly studied area [1].

In their recent article, Prunet and coworkers assessed myocardial glucose metabolism and sympathetic innervation in SAH by means of scintigraphy, and investigated the possible associations with left ventricular (LV) systolic and diastolic dysfunction, troponin elevation and SAH outcomes [1]. The authors provide scintigraphic evidence of impaired glucose metabolism (assessed by ^18^ F-fluorodesoxyglucose positron emission tomography) and sympathetic innervation (studied by ^123^I-meta-iodobenzylguanidine scintigraphy) in 83.3% (25/30) and 90% (27/30), respectively, of 30 patients with acute SAH. Despite extensive changes in glucose metabolism and innervation, coronary perfusion was not affected. The topography and extent of metabolic and innervation scintigraphic defects were largely overlapped, supporting the hypothesis that metabolic shifts occurred secondarily to neurocardiogenic injury rather than cardiac ischemia [2,3]. Follow-up studies showed improvement of glucose metabolism in 50% (15/30) of SAH patients in 1 to 2 months. Normalization of sympathetic innervation was slower, however; only 26.7% (8/30) exhibited normal ^123^I-meta-iodobenzylguanidine scintigraphy 6 months later [1]. These data provide novel insight into the metabolic consequences and their evolution over time in SAH, an issue that was until now largely undetermined.

SAH-induced neurocardiogenic injury demonstrates comparable scintigraphic features [4,5] with TTC, which is also attributed to massive release of catecholamines [6]. However, the topography of these regional abnormalities differs significantly between SAH, which diffusely affects LV myocardium [1], and TTC, in which LV apical myocardium is predominantly affected [4,5]. Furthermore, myocardial scintigraphic defects showed no association to echocardiography in SAH [1], whereas impaired glucose uptake in TTC is closely related with LV apical ballooning on echocardiography.

Although increased adrenergic tone has been accounted for in both SAH and TTC [6], there might be several factors that may explain, at least in part, potential differences in their scintigraphic, echocardiographic and clinical manifestations. Such parameters might regard the culprit catecholamines (directly released noradrenaline in SAH versus circulating epinephrine in TTC) [7] and the adrenoreceptors involved (β1-receptors in SAH versus β2-receptors highly concentrated in the apex in TTC) [8], the nature of myocyte injury (direct cardiotoxicity in SAH [6] versus anti-apoptotic adaptation in TTC [8]), the presence of myocardial ischemia (not validated in SAH [1,9] versus possibly impaired microcirculation in TTC [10]), new polymorphisms and genetic causes of the sympathetic nervous system overactivity [11].

What is remarkable in the study by Prunet and colleagues is not the detection of neurometabolic myocardial alterations in SAH – such changes might probably be expected, since similar findings have been described in TTC. No, what is remarkable is that regional scintigraphic defects may widely exist within myocardium of SAH patients without clinical, echocardiographic or laboratory evidence of neurogenic stunned myocardium (NSM). The term NSM has been introduced to describe myocardial injury and dysfunction occurring after diverse types of acute brain injury (including SAH) as a result of imbalance of the autonomic nervous system [12]. The diagnosis of NSM is based on a wide spectrum of cardiac abnormalities, including LV systolic and diastolic dysfunction, myocardial necrosis and arrhythmias [12]. In this respect, the study by Prunet and coworkers widens the existing knowledge, suggesting that neurometabolic cardiac abnormalities in acute SAH may represent a novel, subclinical type of NSM – extending beyond the classic NSM, as the latter has been defined until now.

One should point out that the presence of neurometabolic cardiac abnormalities showed no association with clinical outcomes in Prunet and colleagues’ study [1]. However, we previously showed that depressed LV systolic function in acute SAH may adversely influence outcomes [13]. Our results were confirmed by more recent data [14]. It is intriguing to speculate that a higher degree of catecholamine-induced cardiotoxicity is probably required for neurometabolic NSM to switch into mechanical NSM and acute heart failure [7,15]; however, this is merely a hypothesis that needs to be studied.

In conclusion, the study by Prunet and colleagues underlines two different aspects in SAH. First, alterations in glucose metabolism, possibly neurogenic in origin, may affect widely the myocardium during the acute phase of SAH. Second, and more important, the study emphasizes that prolonged and slowly reversible neurometabolic abnormalities may globally occur in SAH even when conventional cardiac evaluation is negative for NSM. The clinical impact of such neurometabolic shifts may be greatly underestimated, as they are difficult to diagnose in the clinical practice. Additional research, including also hemodynamically unstable patients with depressed LV function, is necessary to clarify further the role of neurometabolic cardiac alterations in acute SAH.

## Abbreviations

LV: Left ventricular
NSM: Neurogenic stunned myocardium
SAH: Subarachnoid hemorrhage
TTC: Takotsubo cardiomyopathy
